# Molecular tools and genetic markers for the generation of transgenic sexing strains in Anopheline mosquitoes

**DOI:** 10.1186/s13071-018-3207-8

**Published:** 2018-12-24

**Authors:** Federica Bernardini, Roya Elaine Haghighat-Khah, Roberto Galizi, Andrew Marc Hammond, Tony Nolan, Andrea Crisanti

**Affiliations:** Department of Life Sciences, Imperial College London, South Kensington Campus, London, SW7 2AZ UK

**Keywords:** Malaria, *Anopheles* mosquitoes, sex determination, genetic engineering, vector control

## Abstract

Malaria is a serious global health burden, affecting more than 200 million people each year in over 90 countries, predominantly in Africa, Asia and the Americas. Since the year 2000, a concerted effort to combat malaria has reduced its incidence by more than 40%, primarily due to the use of insecticide-treated bednets, indoor residual spraying and artemisinin-based combination drug therapies. Nevertheless, the cost of control is expected to nearly triple over the next decade and the current downward trend in disease transmission is threatened by the rise of resistance to drugs and insecticides. Novel strategies that are sustainable and cost-effective are needed to help usher in an era of malaria elimination. The most effective strategies thus far have focussed on control of the mosquito vector. The sterile insect technique (SIT) is a potentially powerful strategy that aims to suppress mosquito populations through the unproductive mating of wild female mosquitoes with sterile males that are released *en masse*. The technique and its derivatives are currently not appropriate for malaria control because it is difficult to sterilise males without compromising their ability to mate, and because anopheline males cannot be easily separated from females, which if released, could contribute to disease transmission. Advances in genome sequencing technologies and the development of transgenic techniques provide the tools necessary to produce mosquito sexing strains, which promise to improve current malaria-control programs and pave the way for new ones. In this review, the progress made in the development of transgenic sexing strains for the control of *Anopheles gambiae*, a major vector of human malaria, is discussed.

## An evolutionary perspective of sex and sex determination in insects

Reproduction is a fundamental feature for life to subsist. In the majority of eukaryotes, sexual reproduction has evolved to allow, through meiosis and fusion of gametes, the rapid appearance of new genetic traits [1]. Sex-specific life histories and adaptations can result in highly specialised sexes that act to maintain this reproduction strategy [2–4].

A common feature of sex determination is a signalling cascade that begins with a singular primary signal and results in the expression of several hundred sexually dimorphic traits, often via a set of sex-specifically spliced intermediates. In the model organism *Drosophila melanogaster*, sexual development is traditionally thought to be initiated by the balance of female determinants on the X chromosome and male determinants on the autosomes. A more recent work supports the alternative view that the primary sex determining signal in flies is represented by the number of X chromosomes in the embryo rather than the X:A ratio [5]. Around 2 hours after fertilisation, the primary signal sets the state of activity of the sex lethal (*sxl*) gene. Sxl is itself a splice factor that acts upon the transformer (*tra*) transcript to produce a female-specific form of the mRNA that encodes a functional Tra protein. In turn, Tra triggers splicing of doublesex (*dsx*) pre-mRNA resulting in expression of the female-specific isoform, Dsx^F^ protein. In its capacity as a transcription factor, Dsx^F^ leads to the development of females. In the absence of Sxl protein, the primary transformer transcript is spliced to produce an mRNA that does not encode functional Tra protein. This consequently determines splicing of the *dsx* primary transcript to produce a Dsx^M^ transcription factor that enable masculine features [6]. In other organisms, sex determination is due to a trans-acting male-determining gene located on the male sex chromosome, generally identified as the Y. When this gene is expressed, during early embryogenesis, it inhibits factors that destine the sex determination pathway to the default female form, thus determining male development [7]. In the organisms that fall into this category, unlike the situation in *Drosophila*, the presence of the male chromosome is a crucial factor for determining sex.

*Anopheles gambiae* is a member of the *Anopheles gambiae* complex that includes the principal vectors of human malaria [8]. Due to the burden imposed by these mosquitoes, efforts have been made to characterise their biology and special attention has been given to sex determination. In 1979 Baker *et al*., found that triploid *Anopheles culifacies* individuals with 3 X chromosomes were phenotypically females whereas XXY individuals showed a male phenotype [9], thus indicating that in mosquitoes the primary signal is different from that which is observed in the *Drosophila* model. In addition, a number of experiments in several *Anopheles* species used male inheritance of translocations as evidence pointing to the involvement of the Y chromosome in sex determination [10–13]. Recently, Krzywinska et al., isolated and characterised a gene, *Yob*, which acts as a male determination factor in *An. gambiae* [14]. This gene is located on the Y chromosome and controls male-specific splicing of *dsx*, the only downstream member of the sex determination cascade known in anopheline mosquitoes [15]. In the Asian malaria vector *An. stephensi*, a small protein named GUY1 acts as a primary signal that affects embryonic development in a sex-specific manner. This protein is encoded by a Y-linked gene, *Guy1*, that represents the best candidate for the male-determining factor [16]. In *Aedes aegypti*, *Nix*, a dominant male-determining factor located within a Y chromosome-like region called M locus, has been recently identified [17].

## Transgenic sexing strains for vector control

Vector control is a crucial component of many disease control programmes. Sterile Insect Technique (SIT) is a species-specific and environmentally non-polluting method of insect control which aims to reduce the ability of the targeted species to produce viable offspring [18]. This technology requires repeated releases of mass reared male insects that are generally sterilised using irradiation. By competing with wild males and mating with females over time, sterilised males lead to reduction of the targeted insect population. SIT programs have been successfully used for the control or local elimination of some insect pests such as the tsetse fly [19], medfly [20], melon fly [21] and the screwworm [22].

A major drawback for the application of this technique to mosquitoes is the reduced reproductive competitiveness of radiation-sterilised strains [23, 24]. This contributed to the failure of SIT programs against *Anopheles quadrimaculatus* in Florida [25, 26] and *Culex tritaeniorhynchus* in Pakistan [27]. In El Salvador, chemosterization was used as an alternative to induce sterility in *Anopheles albimanus* with promising results [28], though the toxicity of the chemical’s residue was not acceptable for large-scale use. Improvements in mass rearing techniques and irradiation sterilisation, coupled with thorough investigations of important issues, such as gamma ray dosage and male fitness parameters, led to the successful demonstration of population suppression of *Aedes albopictus* in northern Italy [29]. However, the technique and its derivatives remain inappropriate for malaria control, due to the difficulty to sterilise males without compromising their ability to mate and the lack of a reliable method for mosquito sex separation. The latter is crucial, as the accidental release of female mosquitoes would contribute to disease transmission.

For other technologies, such as Incompatible Insect Technique (IIT), female-free releases are also fundamental [30, 31]. IIT strategies are shown to be valuable for the control of various mosquito species [32, 33]. In these IIT methods, sterility is induced by *Wolbachia*, which is an obligate intracellular bacterium. When infected male mosquitoes mate with uninfected females or with females infected with incompatible *Wolbachia* strains, the microorganism induces cytoplasmic incompatibility, a phenomenon that results in embryonic lethality. Accidental releases of female mosquitoes infected with the *Wolbachia* strain used for the sterilisation program would interfere with the effectiveness of IIT and could potentially cause its failure.

Irradiation and classical genetics have been exploited to generate efficient genetic sexing strains in a number of tephritid species [21, 34–37]. A genetic sexing strain for *Anopheles arabiensis* mosquitoes has been established based on dieldrin resistance; however, there are some concerns over the use of this strain for large scale application that revolves around the use of the insecticide [38]. Other methods based on mechanical, behavioural and developmental approaches have been developed, however, the natural variation of parameters used for sex separation, particularly high for some mosquito species, poses a limit to their relatability [39]. The generation of transgenic sexing strains where inherited traits, such as fluorescent markers or resistance to drugs, are specifically associated with one of the two sexes could be used as a high throughput method for mosquito sex separation and facilitate the releases of male-only mosquitoes for SIT based approaches.

An alternative strategy based on the knowledge of the molecular mechanisms involved in the sex determination pathway, has been successfully exploited to develop a transgene-based female-specific lethality system for early embryonic sexing in medfly. The system relies on the sex-specifically spliced *transformer* intron to restrict the expression of a lethal gene only in females [40]. In *An. gambiae*, injections of the male-determining factor, *Yob*, into young embryos are lethal to females [14]. Similarly, in *An. stephensi*, ectopic expression of *Guy1* confers 100% female lethality during embryonic and early larvae stages [41]. Ectopic expression of *Nix* in *Ae. aegypti* gives masculinized females [17]. This female-killing property could be used to eliminate harmful female mosquitoes or convert them into harmless males.

A similar principle led to the development of the sex-ratio distortion technique in mosquitoes. In 2014, Galizi et al. generated a transgenic *An. gambiae* strain expressing the endonuclease IPpo-I under the testes-specific *ß2*-tubulin promoter. IPpo-I recognises and cuts ribosomal rDNA repeats located in a single cluster on the *An. gambiae* X chromosome. Upon IPpo-I activity, only Y chromosome-bearing sperm remain functional, enabling transgenic males to generate only male progeny [42]. A similar result was achieved using a CRISPR-Cas9 sex distortion system [43]. If the sex-ratio distorter is linked to the Y chromosome, this technique has the potential to quickly spread and suppress a wild population even if a very low number of male mosquitoes are released [44, 45]. Unfortunately, the biology and complex structure of the *Anopheles* Y chromosome has hampered progress in this field and restricted the expression of the sex-ratio distorter from an autosomal location.

Mosquito sexing strains are urgently needed for the improvement of existing malaria control strategies and for the development of new ones [46]. In the following paragraphs progress toward the generation of sexing strains using transgenic methods is reviewed.

## Genetic engineering for the generation of transgenic sexing strains

### Transposase induced integration

Nearly 70 years ago, McClintock identified Transposable Elements (TE), which are DNA sequences that are able to change their position within the genome [47]. TEs can be manipulated to allow the insertion of foreign DNA into a genome and their use has been implemented in a variety of research fields [48–53]. The first successful germline transformation system in insects was based on the *Ρ-*element transposon naturally present in the *D. melanogaster* genome [54]. Subsequently, TEs such as *piggyBac* (PB) and *Tc1/mariner* transposons have been characterised in both prokaryotes and eukaryotes where they are typically present in large numbers [55]. Although the first *Anopheles* transgenic strain was generated using a transposon belonging to the *Tc1/mariner* family [56], the most widely used system for germline transformation in this organism is PB-based transposition [57, 58].

DNA susceptibility to transgene integration varies throughout the genome. This is particularly true when it comes to the Y chromosome. The *An. gambaie* Y chromosome comprises 10% of the genome and is estimated to be around 26 megabases in size. The accumulation of repetitive sequences, many of which are also present on other chromosomes, has made the assembly of Y chromosome sequences difficult. To date, only 5 genes have been identified on the Y chromosome that is mainly constituted by satellite DNAs and transposons [59]. Given its small size and chromatin structure, it is not surprising that PB integrations onto the *Anopheles* Y chromosomes are rarely reported. It has been speculated that because of its heterochromatic nature, the Y chromosome could be refractory to the random integration catalysed by transposases. In addition, the regulatory regions commonly used to express marker genes to identify transformation events could be subjected to transcriptional silencing when they land on the Y chromosome. Nevertheless, in 2014, Bernardini et al. characterised an *An. gambiae* strain carrying a transgene on the Y chromosome. The transgenic strain was generated through microinjections of wild-type *An. gambiae* embryos with a plasmid containing PB inverted repeats flanking the 3xP3-GFP and Actin5C-RFP fluorescent markers. The neuronal promoter 3xP3 leads to strong expression of the GFP from 1^st^ instar larval stage, while Actin5C promoter displays patchy RFP visible mainly in the periphery of the larval gut. In addition, the transgene contains an I-SceI endonuclease recognition site. As a result, only the transgenic male mosquitoes expressed the fluorescence pattern while females were negative for fluorescence [60].

As emphasised above, reliable systems for sex separation of mosquitoes is of paramount importance. Manual and automated sex separation systems based on transgenic male mosquitoes have been previously reported for *An. stephensi* [61]. This method relies on the presence of fluorescent markers driven by the male specific *ß2*-tubulin promoter. Under this condition, fluorescent markers are expressed in the gonads around 3^rd^ instar larval stage. The strong expression of Y-linked fluorescent markers in early stages reduce larval mortality or fitness impairment due to sorting procedures and facilitate sex separation methods. Furthermore, early selection minimises the overall number of larvae reared in laboratory conditions thus reducing operational costs. To explore this opportunity, the transgenic strain carrying Y-linked fluorescent markers was used to separate *An. gambiae* males and females using a Complex Object Parametric Analyser and Sorter (COPAS). Multiple experiments, where over 16,000 larvae were processed for several generations, led to the accurate separation of fluorescent positive males from fluorescent negative females demonstrating the utility of the Y-transgenic strain for high-throughput mosquito sex separation [62].

Marois et al. also showed that a large number of non-transgenic *An. gambiae* males can be isolated by COPAS if the fluorescent marker is linked to the X-chromosome. In this case, hemizygous males, carrying the X-linked transgene were crossed to wild type females and their progeny sorted into transgenic females and non-transgenic males [63].

A different embryo injection experimental method led to the generation of an independent *An. gambiae* Y-linked transgenic strain (Galizi et al., unpublished). Once again, Y integration of the transgene was identified by observation of male-only inheritance of the fluorescent markers. To date, the Y strains described here represent the only examples of *An. gambiae* mosquitoes carrying transgenes on the Y chromosome. However, the increased knowledge of the Y chromosome content together with the fast development of molecular tools for genetic engineering might help the generation of transgenic male sexing strains.

### φC31-att induced site specific integration

As previously described, the Y-transgenic strain characterised by Bernardini et al. carries an I-SceI recognition site [60]. The exclusive presence of this site in the transgenic construct facilitated the genetic engineering of the Y chromosome. Targeted knock-in by homology-directed repair was used to modify the transgene and insert an *attP* recombination signal onto the Y chromosome. The *attP* is recognised by the phage φC31 integrase and allows site-specific integration of DNA constructs carrying a corresponding *attB* sequence [64, 65]. This transgenic line offers an alternative to the use of fluorescent markers for high throughput mosquito sex separation. In fact, selectable markers could be specifically inserted onto the Y chromosome thus conferring different features to males and females. For example, SIT programs for the control of the Mediterranean fruit fly *Ceratitis capitata* make use of genetic sexing strains containing a Y-linked translocation that rescues a temperature sensitive lethal mutation only in males [66]. The *An. gambiae* Y-linked *attP* strain allows for the insertion of such a gene directly onto the Y chromosome overcoming fitness reduction due to mutagens used to induce translocations. Alternatively, to achieve sex separation in rearing conditions, Y-linked genes conferring resistance to an insecticide could be used [10, 38, 67–70].

As a number of vector control strategies aim to deliver genes of interest specifically onto the Y chromosome, the Y-attP line represents a promising tool for malaria vector control. For example, site-specific integration onto the Y chromosome would benefit the efficiency of techniques such as sex distortion [42]. In fact, a sex-distortion system targeting the X chromosome would be inherited by all the male progeny when integrated onto the Y chromosome, allowing the transgene to quickly spread into the mosquito population [44, 45].

### CRISPR-Cas induced homology-directed insertion

**Fig. 1:**
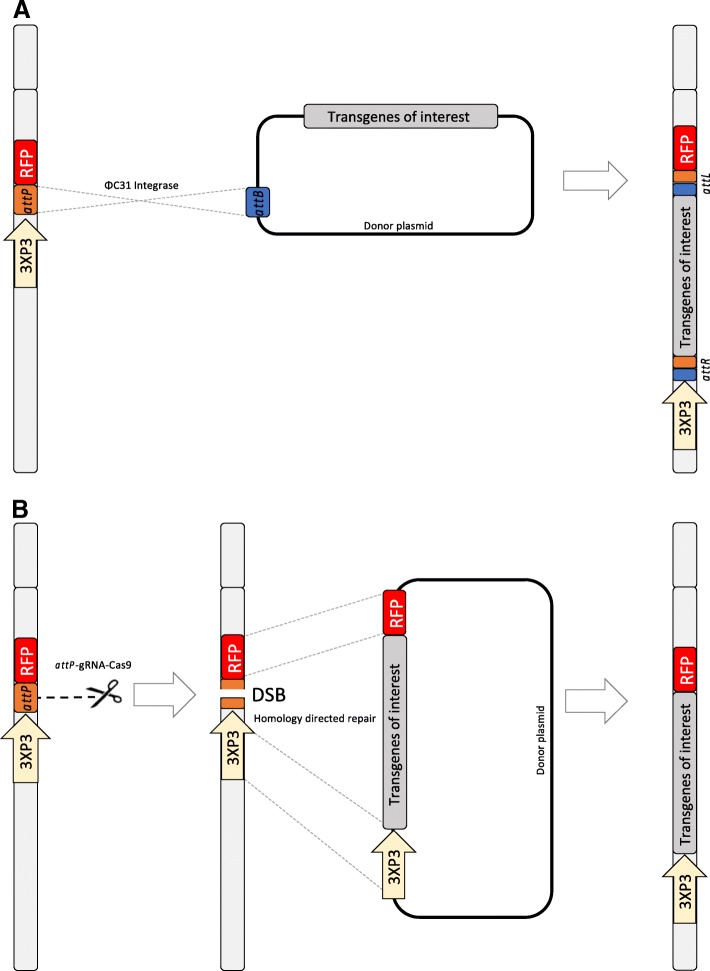
Schematic view of site-specific integration of transgenes into the Y chromosome of the Anopheles gambiae Y-attP strain. The Y-attP line carries the attP docking site and a 3xP3-RFP fluorescence marker transcription unit [60]. **a** The ΦC31 integrase, provided in the form of plasmid, catalyses the recombination reaction between the attP target sequence (orange) and attB donor sequence (blue) resulting in the site-specific integration of the transgenic construct onto the Y chromosome. After recombination, hybrid sites called attL and attR are generated, which are no longer recognised by the integrase thus conferring stability to the integration. **b** A Cas9 coupled with a gRNA (shown as the molecular scissors) induces a double-strand break (DSB) at the attP target site. A donor plasmid containing homologous regions upstream and downstream of the DSB site acts as a template for homology-directed repair. This results in the insertion of the transgenes of interest into the Y chromosome (Table 1; Haghighat-Khah RE et al., unpublished)

Since 2012, the Clustered Regularly Interspaced Short Palindromic Repeats (CRISPR)/ CRISPR associated gene (Cas) system (CRISPR/Cas or CRISPR) has simplified the process of highly-specific genome engineering [71]. In its simplest form, a single endonuclease called Cas9 is directed to its genomic target by a single ‘guide’ RNA molecule that has approximately 20 base-pair complementarity to its DNA target. Recently, the technology has been adapted for site-specific knock-in *An. stephensi* [72] and *An. gambiae* [73]. Recent evidence suggests that the strategy may be far more efficient than recombinase-mediated integration. In a proof-of-principle experiment (Haghighat-Khah, R.E. et al., unpublished), CRISPR-Cas induced knock-in was successfully used to modify the *An. gambiae* Y chromosome (Fig. 1). The CRISPR guide RNA (gRNA) was designed to target the *attP* sequence present in the Y-attP transgenic line described above. Further, despite a small sample size, results suggested that the CRISPR-cas9 system was more efficient than site specific integration φC31 mediated, producing more transgenic G0 pooled cages than insects injected using φC31 (Table 1). This is the first reported use of CRIPR-cas9 system to modify the Y chromosome of any organism. The CRISPR-cas9 system provides a method to edit almost any chosen DNA sequence without requiring complex protein engineering and selection procedures. Therefore, this precise and efficient transformation technology can be used to insert sequences into any Y-linked loci such as those recently identified by Hall et al*.* [59]. This has widespread implications for further developments of any male-biased transgenic system, which could be inserted into any transcriptionally active region on the Y by redesigning the gRNA target site. The system could also be used to modify the *An. gambiae* Y-attP line to include carefully positioned *LoxP* or *FRT* sites to enable iRMCE (integrase-Recombinase mediated cassette exchange) [74]. This would expand the molecular toolbox for modifying the *Anopheles* Y and allow for the direct comparison and characterisations of components such as lethal, suppressor or refractory effector elements to fine-tune expressed phenotypes.

**Table 1:** Comparison of integrase-based and nuclease-based approaches to engineer the Y-chromosome of *Anopheles gambiae*

Donor plasmid	Helper Plasmid	Injected G0 embryos	Surviving G0 larvae	Transient G0 Larvae	Transient G0 adult ♂	Pooled cages	Transgenic G1s
A							
33 nM pHomeT	35 nM	1769	134	44	29	5	16/6559
	ϕC31 integrase						1 pooled G0 transient cage
33 nM pHomeT	35 nM	600	127	68	29	6	63/5147
	*attP*-gRNA						3 pooled G0 transient cages
B							
40 nM attBCFP-VasaGFP	96 nM	~2000	n/a	n/a	5	n/a	6/791
	ϕC31 integrase						
52 nM 3xP3[AttP]RFP	90 nM	~4000	n/a	n/a	15	n/a	11/6160
	I-SceI						

**(A)** ΦC31-att recombinase was used to insert a vector, pHomeT, containing the attB donor sequence into the Y-attP strain, carrying the complementary attP target sequence on the Y-chromosome. CRISPR-cas9-directed knock-in was used to integrate the pHomeT plasmid into the same Y locus. **(B)** ΦC31-att recombinase was used to integrate a vector, attBCFPVasaGFP, containing the attB donor sequence into the Y-attP strain. I-SceI-directed knock-in was used to integrate the vector 3xP3[AttP]RFP into the Y chromosome of T4 strain, carrying the I-SceI recognition site [60].

### Transgenic sexing strains for genetic introgression

The possibility to specifically label sex chromosomes assumes importance for experiments based on classical genetics. In this context, it is often important to track the identity of chromosomes through genetic crosses for successive generations. Given the lack or reduced recombination of sex chromosomes in *An. gambaie* [75–78], transgenic sexing strains can be used for genetic introgression across sibling mosquito species.

*Anopheles arabiensis* is another important vector of malaria. Rearing this species in lab settings is widely known to be challenging and genome sequences have not yet been assigned to chromosomes [79, 80]. Despite the medical importance of this species, there has been little progress in the application of molecular tools for its DNA modification, and to date no transgenic strain is available. Recently, Bernardini *et al* described a number of genetic crosses that allowed the introgression of the *An. gambiae* Y chromosome into the *An. arabiensis* genetic background [62]. For this purpose, the previously described *An. gambiae* Y-transgenic strains carrying Y-linked fluorescent markers were used, allowing the identification of the *An. gambiae* Y through many generations. Y chromosome introgression was confirmed by both genetic tests and DNA sequencing. *Anopheles arabiensis* males carrying the *An. gambiae* Y chromosome showed fitness comparable to that of the wild type *An. arabiensis* males, with no decrease in fertility. This experiment gave an insight into the biology of the Y chromosome and its role in reproductive isolation. Further, the introgressed strains carrying the *An. gambiae* Y-linked *attP* and transformation markers are suitable for site-specific integration, mosquito sex separation and could also be used for the application of vector control strategies as discussed for *An. gambiae*.

## Conclusion

Almost 20 years after the first *Anopheles* transgenic strain was generated, substantial progress has been made in the field of transgenic technology. This, coupled with the availability of fine molecular tools for genome editing and an increased knowledge of mosquito genetics and biology, has facilitated significant strategic improvements in the research field that aim to vector control. The recent availability of *Anopheles* strains carrying fluorescent markers or sequences for site-specific integration into the Y chromosome has revolutionised our ability to separate males from females, which was previously based on inefficient procedures such as differences in the size of pupae. The transgenic sexing strains discussed in this review fulfil the requirements of high-throughput mosquito sex separation without affecting mating competitiveness, which is crucial for making population suppression technologies based on sterile male releases technologies much more powerful. In addition, the Y-linked transgenes are inherited by all-male progeny and no segregation of the sexing markers occur, thus facilitating the maintenance of a pure breeding stock in lab conditions. The potential to introduce a genetic trait specifically onto the Y chromosome allows for the generation of a high throughput sorting method based, for example, on insecticide resistance. Furthermore, a Y-linked male-biased trait could be tailored to benefit novel malaria genetic control programs based on sex-ratio distortion techniques. Finally, the sustainable and effective control of malaria is achievable if the strategies discussed here are advanced through a series of feasibility, safety, efficacy and acceptability studies through combined scientific and social discovery and development efforts. This is the basis for the successful application of transgenic vector control in the field to win the fight against malaria.
